# Beneficial Effects of Putative Hydrogen Sulfide (H_2_S) Donor POM16 in a Genetic Model of Amyotrophic Lateral Sclerosis, FUS [1-359]-Transgenic Mice

**DOI:** 10.3390/molecules31173021

**Published:** 2026-08-28

**Authors:** Tatyana Strekalova, Anna Gorlova, Johannes P. M. de Munter, Maya Chervinskaya, Alexey Deykin, Zhanna Aladysheva, Elisaveta Grigorieva, Alexei Lyundup, Sholpan Askarova, Andrey Kostin, Igor Pomytkin

**Affiliations:** 1 Department of Psychiatry and Neuropsychology, School for Mental Health and Neuroscience, Maastricht University, 6229 ER Maastricht, The Netherlands; h.demunter@neuroplast.com; 2 Research and Education Resource Center, Peoples Friendship University of Russia (RUDN University), 117198 Moscow, Russiamachervinskaya@edu.hse.ru (M.C.); grigorieva.es@phystech.edu (E.G.); liundup-av@rudn.ru (A.L.); kostin-aa@rudn.ru (A.K.);; 3 Laboratory of Genetic Technology and Gene Editing for Biomedicine and Veterinary, National Research Belgorod State University (NRBSU), 308015 Belgorod, Russia; alexei@deikin.ru; 4 Department of Normal Physiology, Sechenov First Moscow State Medical University, 119048 Moscow, Russia; aladysheva_zh_i@staff.sechenov.ru (Z.A.); shaskarova@nu.edu.kz (S.A.); 5 Center for Life Sciences, National Laboratory Astana, Nazarbayev University, Astana 010000, Kazakhstan

**Keywords:** amyotrophic lateral sclerosis (ALS), hydrogen sulfide (H_2_S), (2S)-2-aminopentanethioic S-acid, FUS [1-359]-transgenic mice, neuroinflammation, oxidative stress

## Abstract

Amyotrophic lateral sclerosis (ALS) is a fatal neurological disorder characterized by rapid motoneuron degeneration. Hydrogen sulfide (H_2_S), a signaling molecule that regulates post-translational modification, has recently been implicated in the pathophysiology of ALS. Our study aimed to design, synthesize, and investigate the potential effects of (2S)-2-aminopentanethioic S-acid (POM16), an isomer of the slow-releasing H_2_S donor thiovaline, in a genetic ALS model. FUS [1-359]-tg mice, which recapitulate ALS syndrome, and their wild-type (WT) littermates received POM16 (at a dose of 50/mg/kg) or standard ALS therapy riluzole (at a dose of 8 mg/kg/day) dissolved in drinking water, or vehicle, for six weeks starting at nine weeks of age. The onset of paralysis, physiological and motor functions, muscle atrophy, density of spinal cord motoneurons, gene expression of proinflammatory cytokines interleukin-1β (IL-1β) and tumor necrosis factor (TNF), and concentration of oxidative stress marker malondialdehyde (MDA) in the spinal cord were studied. POM16-treated mutants displayed significant improvements in body weight, water and diet intake, as well as behavior in the rotarod, wire, and pole tests. The percentage of mice with paralysis on the 6th week of dosing was reduced from 48% in vehicle-treated mutants to 16% in POM16-treated FUS [1-359]-tg mice, while in the riluzole-treated group, it was 38%, not reaching significance. Notably, muscle weight was not significantly improved by the latter treatment, unlike the dosing with POM16. In comparison with vehicle-treated FUS [1-359]-tg mice, POM16-treated mutants had significantly higher motor neuron density in the spinal cord, lower MDA levels, and reduced muscle atrophy ranking. Thus, new compound POM16 has a therapeutic potential to counteract ALS pathology that is likely mediated via anti-oxidative stress mechanisms. Given that any effective treatment of this devastating disease is currently lacking, it is hoped that POM16 can be a promising therapy for ALS.

## 1. Introduction

Amyotrophic lateral sclerosis (ALS) is a fatal neurodegenerative disease characterized by the progressive degeneration of motoneurons and neurons in the cortex and brainstem, leading to death within 3–5 years after onset [1,2,3]. The etiology of ALS remains largely unclear, and while mutations in over 60 genes have been implicated in its familial or sporadic forms, it is manifested by a narrow spectrum of clinical and pathological hallmarks at advanced disease stages [4,5,6,7]. To date, the treatment of patients with ALS remains a serious problem since only two drugs, riluzole and edaravone, are available, and their clinical efficacy is extremely limited, as their use prolongs life span by 10% in the best case and sometimes can be shown for specific subgroups of patients only [8,9]. As such, the clinical care of patients with ALS primarily focuses on symptom management and quality of life. Thus, the medicinal and social problems of proposing at least a more effective treatment for this detrimental disorder remain very high.

Oxidative stress and neuroinflammation are key contributors to ALS pathogenesis, regardless of the genetic cause of the disease, and are considered important therapeutic targets [10,11]. In recent years, researchers have focused on hydrogen sulfide (H_2_S), a gasotransmitter signaling molecule whose contribution to physiological and pathological processes is thought to be primarily mediated via oxidative stress regulation, acting on L-type Ca^2+^ and KATP channels [12,13,14]. Accordingly, modulation of H_2_S levels can play and important role in ALS mechanisms and potentially, treatment, as has been suggested by recent studies [15,16,17,18,19,20]. The modulation of H_2_S-mediated regulation has attracted considerable attention with regard to the unmet needs of ALS management.

H_2_S regulates physiological processes in mammals through S-sulfhydration, a post-translational modification that converts cysteine thiol (-SH) groups into persulfide (-SSH) groups, thereby regulating protein functions [16,21]. This regulation interferes with cellular metabolism and survival, bioenergetics, oxidative stress, endoplasmic reticulum stress, and inflammation [12,14,22,23]. Endogenous H_2_S is synthesized primarily by three enzymes: cystathionine β-synthase (CBS), cystathionine γ-lyase (CSE), and 3-mercaptopyruvate sulfurtransferase (3-MST) [24]. These enzymes are expressed in a tissue-specific manner, the level of which can be altered distinctly in various diseases, including ALS, where changes in CBS can play a major role [13,19,22]. The concentrations of free H_2_S, its metabolites, and H_2_S–bound forms were shown to be altered in the cerebrospinal fluid (CSF), blood, urine, and breath of patients and animal models with ALS, as well as Alzheimer’s disease, Parkinson’s disease, Huntington’s disease, Down syndrome, multiple sclerosis, autoimmune demyelinating disorders, and others [17,19,25,26,27,28,29].

Notably, both a decrease and an increase in H_2_S levels have been reported to accompany disease conditions [19,26]. Both supplementation and downregulation of H_2_S have been shown to interfere with Alzheimer’s disease, Parkinson’s disease, Huntington’s disease and other disorders [15,19,30], thus suggesting complex mechanisms in which H_2_S is involved. Consequently, the role of H_2_S in health and disease is under debate, and ongoing studies continue to contribute to a better understanding of the therapeutic potential of H_2_S modulation [19,31,32]. Currently, it is commonly believed that low amounts of H_2_S reduce oxidative stress, promote neuroprotection, and counteract inflammation and mitochondrial dysfunction, which is the basis for the therapeutic effects of exogenous supplementation of H_2_S or the use of H_2_S donors [15,32,33,34,35]. High concentrations of H_2_S are considered ‘toxic’ as they disrupt mitochondrial complex IV and mediate glutamatergic neurotoxicity by increasing Ca^2+^ influx [13,36]. Indeed, H_2_S is increasingly considered a double-action regulatory molecule that acts as a neuroprotector at nanomolar to low micromolar doses and as a toxic mitochondrial deregulator at mid-to high-micromolar concentrations [17,37,38,39].

The above-described mechanisms were proposed to underpin the role of H_2_S in ALS, based on the demonstration of increased concentrations of free H_2_S in the CSF of a subset of patients with bulbar-onset form of ALS and in the tissues of SOD1G93A mice, a common ALS model [18,20,22]. Notably, as ALS is characterized by a narrow scope of clinical and pathological hallmarks despite the involvement of a variety of genes and the familial or sporadic nature of the disease [4,5,6,7], it is generally accepted that distinct genetic models of this disorder significantly overlap in recapitulating the cellular and molecular mechanisms of symptomatic ALS [40]. Currently available experimental data concerning the role of H_2_S are limited by the use of SOD1G93A, a genetic model of familial ALS that mimics deficient SOD1 gene functions [18,20]. These studies revealed elevated H_2_S levels in neuronal cell cultures and spinal cords of these mutants [18]. However, other reports suggest that H_2_S provides an antioxidant function through the elevation of H_2_S synthesis enzyme CBS and propose that H_2_S exerts neuroprotective effects in ALS by limiting oxidative modifications and reducing insoluble SOD1 aggregation [22].

Indeed, the neuroprotective action of H_2_S is supported by numerous demonstrations of the beneficial effects of H_2_S donors in various conditions [14,16,24,41]. Impaired H_2_S signaling has been shown to exacerbate amyloid-β and tau pathology and cognitive deficits in the 3× Tg-AD mouse model of Alzheimer’s disease and to contribute to dopaminergic neuron loss, and motor impairment in 6-OHDA- and MPTP-induced models of Parkinson’s disease; clinical studies report altered H_2_S levels in patients suffering from these disorders [19,22,42]. The H_2_S donors sodium hydrosulfide (NaHS) and GYY4137 attenuated both molecular and behavioral abnormalities in animal models of Alzheimer’s and Parkinson’s disease [12,22,34,36]. These data are somewhat discrepant from a recent demonstration of elevated H_2_S levels in the CSF of patients with some of these conditions [19]. Hence, the understanding of the role of H_2_S in CNS disorders is currently quite limited, although the therapeutic use of H_2_S donors represents a potentially promising, albeit underexplored, line of research [17,30,31,32]. Importantly, commonly used H_2_S donors, such as sodium hydrogen sulfide (NaHS) and sodium sulfide (Na_2_S), release H_2_S too rapidly to mimic physiological signaling, limiting their therapeutic applications [41,43].

Notably, thioamino acids, such as sulfur-containing amino acids, thioglycine, and L-thiovaline, are slow-releasing H_2_S donors [43,44]. Thioglycine and thiovaline release H_2_S following catalysis by physiological concentrations of bicarbonate, which is naturally present in blood serum at high concentrations, reaching maximal release between one and eight hours and, thus, might be more appropriate for clinical use [44]. Both thioglycine and L-thiovaline were shown to increase intracellular cyclic guanosine monophosphate (cGMP) levels and elicit vasorelaxation, similar to other H_2_S donors, at matching H_2_S release doses [44]. However, L-thiovaline and thioglycine, which are derivatives of proteinogenic amino acids, are encoded by the genetic code and can be implicated in ribosomal translation during protein synthesis, which may generate toxic effects in vivo [45].

Therefore, in our study, we designed an isomer of the slow-releasing H_2_S donor L-thiovaline, C_3_H_7_CH(NH_2_)COSH, (2S)-2-aminopentanethioic S-acid (POM16), L-thionorvaline, which is considered a non-proteinogenic amino acid because it is not encoded by the genetic code and does not interfere with protein synthesis [45]. This allows for the anticipation of good safety and tolerability compared to L-thiovaline and thioglycine.

A new low-weight water-soluble POM16 compound was designed, synthesized, and studied for stability as well as H_2_S release [46]. Our studies demonstrated the high stability of this compound (see Supplementary File) as well as its properties of slow release of H_2_S (see Supplementary File, Figure S1), which are characteristic of thioamino acids such as thioglycine NH_2_CH_2_COSH and L-thiovaline (CH_3_)_2_CHCH(NH_2_)COSH [47]. Specifically, the use of the fast-responsive S fluoroprobe H_2_S probe P3 based on the reaction between P3 and H_2_S [48] has shown that POM16 significantly increases H_2_S concentrations in an aqueous solution via bicarbonate-catalyzed hydrolysis (Supplementary File, Figure S1), which is typical for slow-releasing H_2_S donors [43,47].

The potential interference of POM16 with ALS-like syndromes was addressed using a genetic model that mimics the second causal to the disease common gene mutation after SOD1, Fused in sarcoma (FUS) gene, FUS [1-359]-tg mice [49,50,51,52,53]. As mentioned above, the cellular, molecular, and biochemical mechanisms of ALS largely overlap in the symptomatic phase of the disease, regardless of the specific gene mutation causing it [54,55,56]. FUS [1-359]-tg mouse line is based on the expression of a truncated human FUS protein lacking RNA-binding capacity, which leads to protein aggregation, oxidative stress, inflammation, mitochondrial dysfunction, and motoneuron death [49,50,51,52]. Mutations in SOD1 cause toxic protein aggregation and accumulation, which converge to a similar scenario of cell function damage, affecting motoneurons as well [1,55]. Both symptomatic SOD1G93A mice and FUS [1-359]-tg mutants display increased brain and spinal cord expression of Iba-1, tumor necrosis factor (TNF), glycogen synthase kinase-3β (GSK-3β), and interleukin-1β (IL-1β), and elevated MDA levels [49,50,51,57,58,59,60,61,62,63,64,65]. A benefit of FUS [1-359]-tg paradigm, compared with other genetic models of ALS, including SOD1G93A mice, is that pathology develops at an early age with abrupt disease onset, in a narrow time window, and rapidly progresses [57,58,66]. This allows for a more accurate detection of the positive or negative effects of various interventions than other models.

To date, the FUS [1-359]-tg model is well established and accepted as a valid animal paradigm of ALS, as it complies with commonly accepted criteria [40], reproducing key pathological and clinical features of the human disease. These hallmarks include the progressive loss of spinal motoneurons, muscle wasting, denervation, asymmetrical limb paralysis, and motor deficits [49,57,58,59,60,66]. Of note, FUS [1-359]-tg mutant mice display increased MDA levels, which can be a target of H_2_S-based therapies. Importantly, interventions with anti-inflammatory and antioxidant properties, including the infusion of human stem cells (“Neuro-Cells”) [58], chronic treatment with anti-inflammatory and antioxidant compounds dibenzoyl thiamine [49,50], and cyclooxygenase-2 inhibition (celecoxib) [57], attenuated these molecular alterations, improved motor outcomes, and slowed disease progression. In addition, standard ALS treatment with riluzole (2-amino-6-trifluoromethoxy-benzothiazole [8]) showed partial efficacy in counteracting ALS-related abnormalities in FUS [1-359]-tg mice, which pharmacologically validates the model [57,58].

Here, we studied whether POM16 interferes with ALS-like syndrome progression in FUS [1-359]-tg mice using previously established readouts [51,57,58,59,60,66]. These readouts included the assessment of the age of disease onset (paralysis), degeneration of spinal motoneurons and muscles, measurement of MDA levels, and gene expression of pro-inflammatory cytokines IL-1β and TNF. Pilot studies using established mouse models of human pathologies demonstrated pronounced beneficial effects of POM16 on behavioral, molecular, and biochemical readouts that were exerted by the 1–3-week administration of this compound in drinking water at doses of 5–30 mg/kg/day, without any signs of toxicity. Since ALS represents a severe syndrome, we chose to use a higher dose of 50 mg/kg/day, at which the dosing for 16 weeks revealed no signs of toxic effects in naïve C57Bl6 mice in the preceding pilot studies.

For pharmacological reference, a standard ALS treatment with riluzole, whose effects have been previously reported in employed-here mutants [8,57,58] and other ALS paradigms [39,67,68] was chosen. The dose of 8 mg/kg/day was selected, based on these preceding studies; higher doses of riluzole did not improve drug efficacy and compromised general behavior and motor symptom evaluation [69]. Riluzole reduces neuronal excitability and glutamatergic excitotoxicity by inhibiting sodium currents and glutamate release, activating the TREK-1/TRAAK channels, enhancing the EAAT2/GLT-1-mediated glutamate clearance, and increasing neurotrophic factors [70,71,72,73,74,75,76,77,78,79]. The reported anti-inflammatory/redox effects are considered secondary to reduced excitotoxicity, rather than direct antioxidant activity [67,80].

As the mechanisms of action of riluzole are supposed to be quite different from those of POM16 and in accordance with the general guidelines of preclinical studies on ALS animal models suggesting the use of standard pharmacological references [40], here we applied previously established treatment regimen with riluzole [57,58].

## 2. Results

### 2.1. Synthesis and Characterization of (2S)-2-Aminopentanethioic S-Acid (POM16)

**Figure 1:**
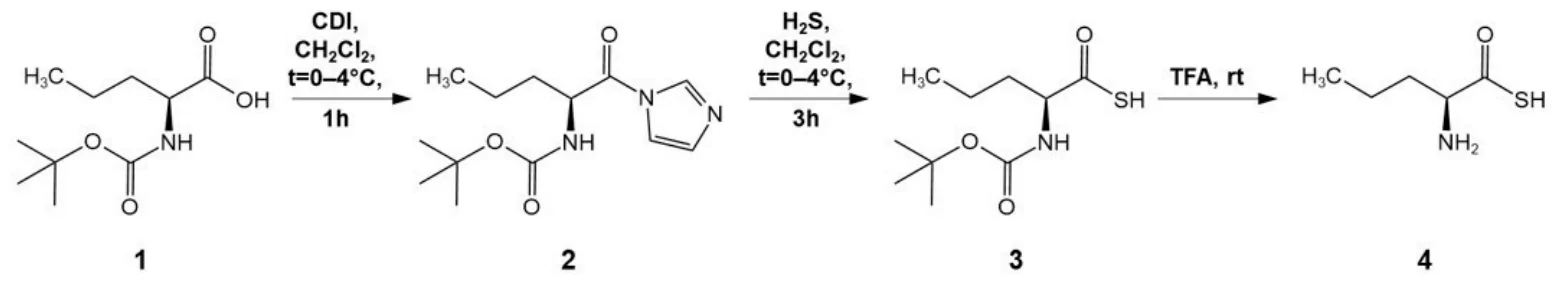
**Synthesis of (2S)-2-aminopentanethioic S-acid (POM16).** Synthesis of POM16 included three steps (see ms text). Compound **1**: *N*-(tert-butoxycarbonyl)-(2S)-2-aminopentanoic acid; compound **2**: 1-[*N*-(tert-butoxycarbonyl)-(2S)-2-aminopentanoyl] imidazole; compound **3**: *N*-(tert- butoxycarbonyl)—(2S)-2-aminopentanethioic S-acid; compound **4**: (2S)-2-aminopentanethioic S-acid (POM16). CDI—carbonyl diimidazole, TFA—trifluoroacetic acid, rt—room temperature.

The synthetic route to POM16 consisted of three steps, starting from the commercially available *N*-Boc-derivative of (2S)-2-aminopentanoic acid 1 (Figure 1).

In the first step, *N*-(tert-butoxycarbonyl)-(2S)-2-aminopentanoic acid (compound **1**, Figure 1) reacts with carbonyl diimidazole (CDI) in methylene chloride at 0–4 °C to obtain 1-[*N*-(tert-butoxycarbonyl)-(2S)-2-aminopentanoyl]imidazole. In the second step, an excess amount of hydrogen sulfide was passed into a solution of compound **2** in methylene chloride at 0–4 °C to obtain *N*-(tert-butoxycarbonyl)-(2S)-2-aminopentanethioic S-acid (compound **3**). Finally, the *N*-Boc protecting group was removed with trifluoroacetic acid to obtain the compound of interest, (2S)-2-aminopentanethioic S-acid (compound **4**, POO16), with a yield of 47% of the starting compound **1**. Compound POM16 was water-soluble at room temperature at high concentrations [46].

### 2.2. Physiological Changes in FUS [1-359]-tg Mice Treated with Riluzole or POM16

**Figure 2:**
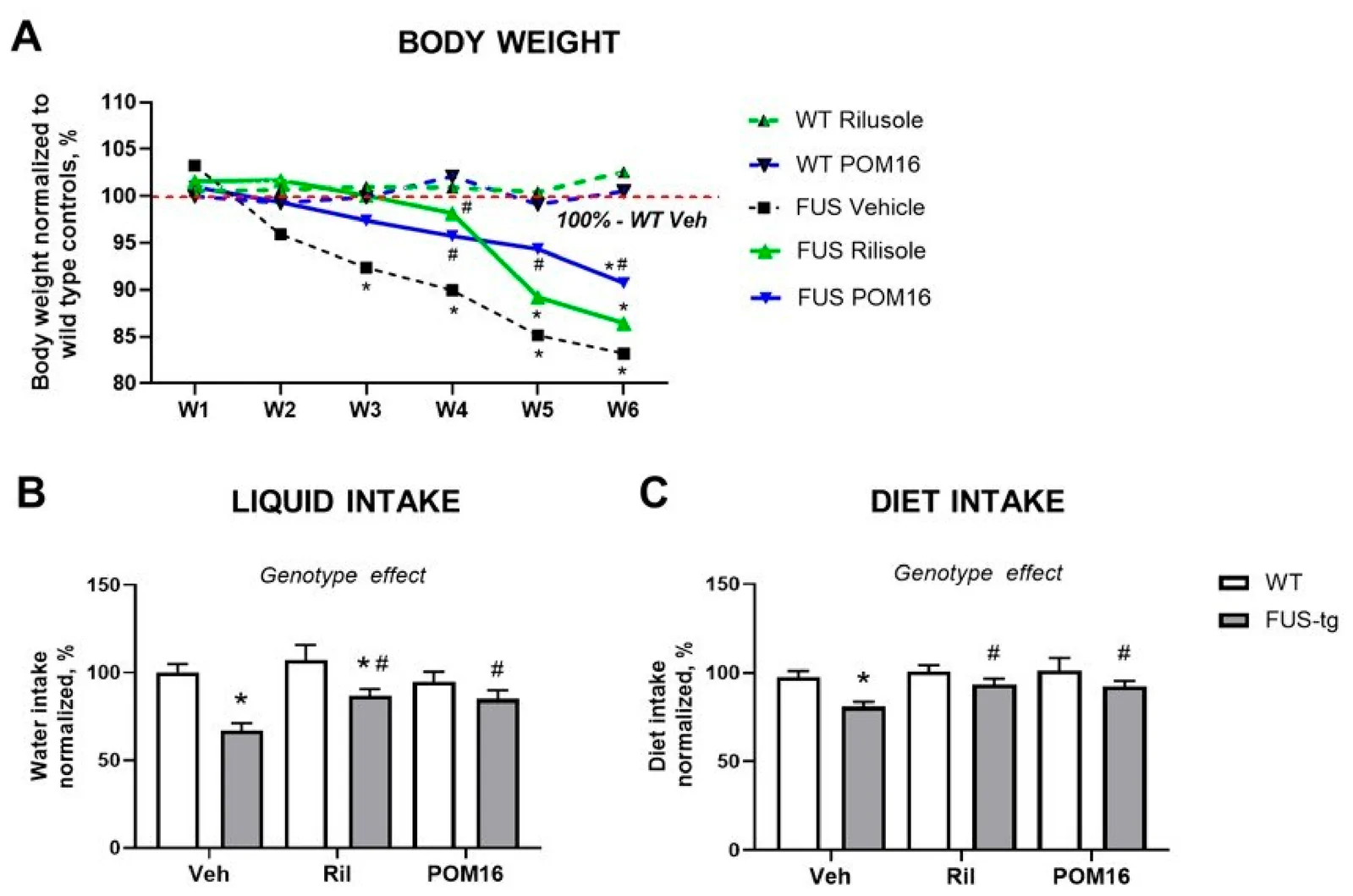
Effects of riluzole and POM16 on physiological parameters in FUS [1-359]-transgenic mice. (**A**) Body weight, (**B**) liquid intake, and (**C**) diet intake were normalized to the WT-Veh group. * *p* < 0.05 vs. respective WT group, # *p* < 0.05 vs. FUS-tg-Veh group. Two-way ANOVA and post hoc Tukey’s test. Each WT group comprised 10 animals; FUS-tg groups: Vehicle, *n* = 21–23; riluzole, *n* = 16; POM-16, *n* = 18–21. Ril—riluzole All data are presented as mean ± SEM.

Two-way ANOVA indicated a significant effect of genotype on body weight, normalized to the means of WT controls (F_20,386_ = 30.12, *p* < 0.0001, two-way ANOVA). Body mass was significantly decreased in FUS [1-359]-tg (FUS-tg) mice that received vehicle in the 3rd-6th weeks compared to that in the WT-Veh group (*p* = 0.029, *p* = 0.033, *p* = 0.012, and *p* = 0.001, respectively; Tukey’s test, Figure 2A). Similarly, FUS-tg-Ril mice displayed significant body weight reduction compared to WT-Veh group on the 5th and 6th weeks (*p* = 0.038 and *p* = 0.024, respectively) and in the FUS-tg-POM16 group on the 6th week (*p* = 0.035; Figure 2A). We also found a significant increase in body weight normalized to the basal values in WT-Veh, WT-Ril, and WT-POM16 mice from week 1 to week 6 (F_20,386_ = 8.07, *p* = 0.023; repeated measures ANOVA and post hoc Tukey’s tests), which indirectly suggests a lack of toxic effects of the treatments used in the experimental groups of control mice.

Two-way ANOVA of normalized liquid intake revealed a significant main effect of genotype (F_1,84_ = 20.82, *p* < 0.0001), whereas no significant effects of treatment (F_2,84_ = 2.736, *p* = 0.0706) or genotype × treatment interaction (F_2,84_ = 2.245, *p* = 0.1123) were found. FUS-tg-Veh and FUS-tg-Ril mice showed significantly lower normalized liquid intake than their respective WT controls (*p* < 0.0001 and *p* = 0.0169, respectively, Tukey’s test; Figure 2B). Both riluzole and POM16 significantly increased liquid intake in mutant mice compared to vehicle-treated mutants (*p* = 0.0111 and *p* = 0.0140, respectively, Tukey’s test; Figure 2B).

### 2.3. Improved Motor Functions in FUS [1-359]-tg Mice That Received Riluzole or POM16

**Figure 3:**
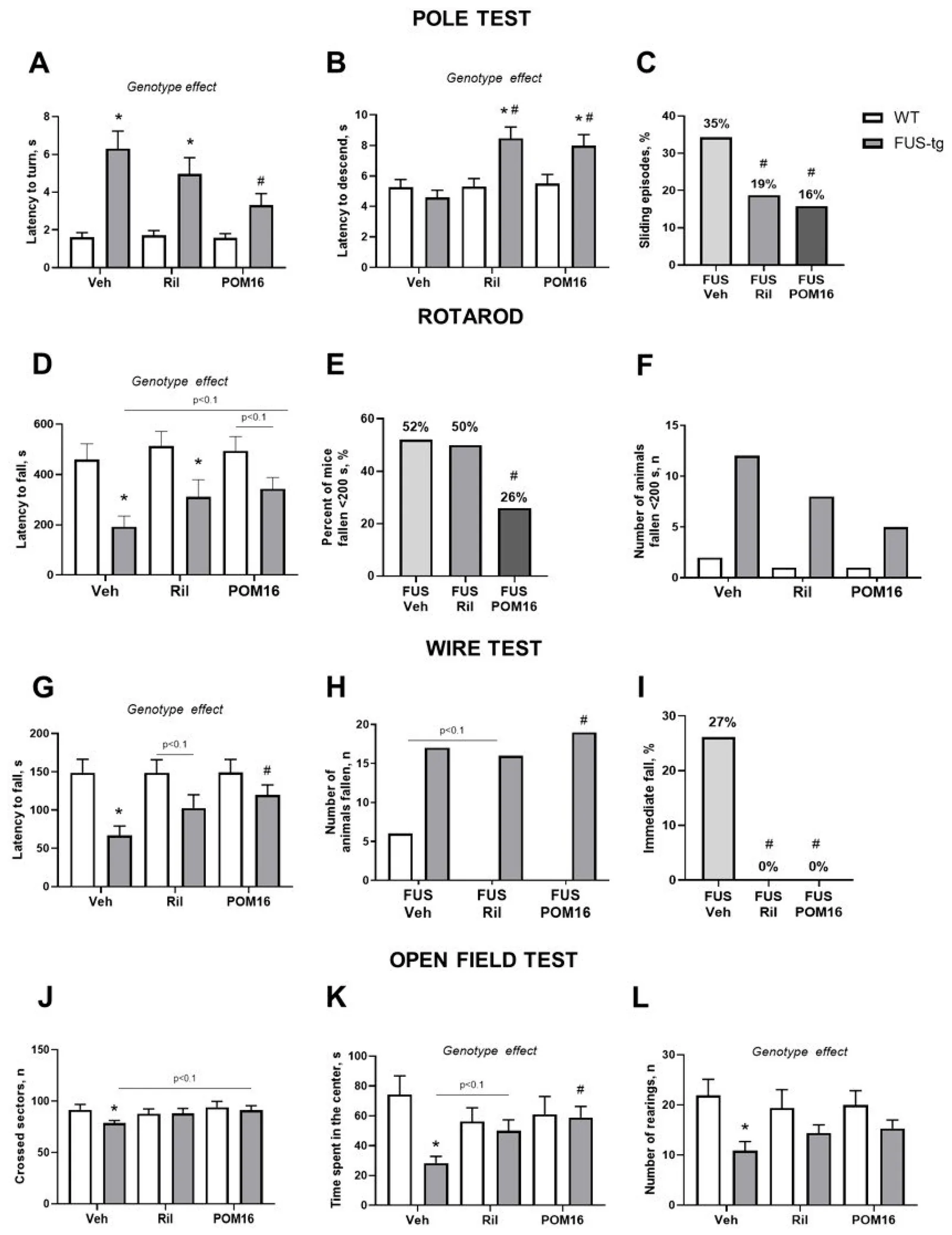
Motor parameters in FUS [1-359]-transgenic mice and effects of riluzole or POM16. (**A**) Latency to turn, (**B**) latency to descend, and (**C**) percentage of sliding episodes in the pole test. (**D**) Latency to fall from the rotarod, (**E**) percentage of mice, and (**F**) number of mice that fell from the rotarod within the first 200 s of the test. (**G**) Latency to fall in the wire-hanging test, (**H**) number of mice that fell during the first 10 s of the test, and (**I**) percentage of mice that demonstrated immediate fall from the wire. (**J**) Number of crossed sectors, (**K**) time spent in the center of the open field, and (**L**) total number of rearing events. * *p* < 0.05 vs. respective WT group, # *p* < 0.05 vs. FUS-tg-Veh group. Two-way analysis of variance (ANOVA), post hoc Tukey’s test, and Fisher’s exact test. Each WT group comprised 10 animals; FUS-tg groups: for rotarod, vehicle, *n* = 23; riluzole, *n* = 10; POM-16, *n* = 19; for pole test, vehicle, *n* = 21; riluzole, *n* = 19; POM-16, *n* = 19; for wire test, vehicle, *n* = 21; riluzole, *n* = 16; POM-16, *n* = 18; for open field, vehicle, *n* = 21; riluzole, *n* = 16; POM-16, *n* = 19. Ril—riluzole. All data are mean ± SEM.

In the pole test, two-way ANOVA of the latency to turn on the vertical bar revealed a significant main effect of genotype (F_1,83_ = 22.74, *p* < 0.0001), whereas the main effect of treatment (F_2,83_ = 1.692, *p* = 0.1905) and the genotype × treatment interaction (F_2,83_ = 1.609, *p* = 0.2062) were not significant. FUS-tg-Veh mice showed a significantly longer time to turn compared to WT-Veh mice (*p* = 0.0001, Tukey’s test). FUS-tg-Ril mice also demonstrated a significant increase in this parameter relative to the WT-Ril group (*p* = 0.0071, Tukey’s test). Notably, FUS-tg-POM16 mice showed a significant reduction in the time to turn compared to the FUS-tg-Veh group (*p* = 0.0066, Tukey’s test; Figure 3A).

Moreover, two-way ANOVA revealed significant main effects of genotype (F_1,80_ = 8.687, *p* = 0.0042) and treatment (F_2,80_ = 5.138, *p* = 0.0080), as well as a significant genotype × treatment interaction (F_2,80_ = 4.491, *p* = 0.0142) in the latency to descend the vertical bar. FUS-tg-Ril mice exhibited a significantly longer time to descend than the WT-Ril group (*p* = 0.0022, Tukey’s test). Similarly, FUS-tg-POM16 mice demonstrated a significant increase in this parameter compared to WT-POM16 mice (*p* = 0.0123, Tukey’s test). Furthermore, both FUS-tg-Ril and FUS-tg-POM16 mice showed a significant reduction in the time to descend compared with vehicle-treated mutants (*p* < 0.0001 and *p* = 0.0001, respectively, Tukey’s test; Figure 3B).

Fisher’s exact test revealed a significant difference in the percentage of sliding episodes during the pole test between the groups (*p* = 0.0035). A significant difference was observed in the pairwise comparison of FUS-tg-Veh and FUS-tg-Ril mice (*p* = 0.0151, Fisher’s exact test), and a significant difference was observed between FUS-tg-Veh and FUS-tg-POM16 mutants (*p* = 0.0028, Fisher’s exact test; Figure 3C). No significant difference was observed in the pairwise comparison of FUS-tg-Veh and FUS-tg-Ril mice (*p* = 0.7773, Fisher’s exact test).

In the rotarod paradigm, two-way ANOVA of the latency to fall from the rotarod demonstrated significant main effects of genotype (F_1,82_ = 18.77, *p* < 0.0001), while the effects of treatment (F_2,82_ = 1.626, *p* = 0.2030) or genotype × treatment interaction (F_2,82_ = 0.4985, *p* = 0.6093) were not significant. FUS-tg-Veh mice exhibited a significantly shorter latency to fall compared to WT-Veh mice (*p* = 0.0013, Tukey’s test), and FUS-tg-Ril mutants showed a significant reduction in this parameter relative to their WT-Ril counterparts (*p* = 0.0204, Tukey’s test; Figure 3D). A trend for a decrease in the latency to fall was observed in the FUS-tg-POM16 animals compared to the WT-POM16 group (*p* = 0.0690, Tukey’s test), although the groups did not differ significantly. Moreover, FUS-tg-POM16 mice had a strong trend for a prolongation in the latency to fall compared to the vehicle-treated FUS-tg mutants (*p* = 0.0631, Tukey’s test).

Fisher’s exact test revealed a significant difference in the percentage of falling episodes during the first 200 s of the rotarod between the groups (*p* = 0.0001). No significant difference was observed in the pairwise comparison of FUS-tg-Veh and FUS-tg-Ril mice (*p* = 0.7773, Fisher’s exact test), whereas a comparison of FUS-tg-Veh and FUS-tg-POM16 mice showed a significant difference (*p* = 0.0002, Fisher’s exact test; Figure 3E). Next, Fisher’s exact test revealed a borderline significant difference in the number of animals that fell from the rotarod during the first 200 s (*p* = 0.0912; Figure 3F). A pairwise comparison of FUS-tg-Veh and FUS-tg-Ril groups, as well as FUS-tg-Veh and FUS-tg-POM16 groups, showed no significant differences (*p* > 0.9999 and *p* = 0.1203, respectively; Fisher’s exact test).

In the wire-hanging test, two-way ANOVA revealed a significant main effect of genotype (F_1,79_ = 15.36, *p* = 0.0002) on the latency to fall from the wire, while the main effect of treatment (F_2,79_ = 1.393, *p* = 0.2544) and the genotype × treatment interaction (F_2,79_ = 1.368, *p* = 0.2605) were not significant. FUS-tg-Veh mice exhibited a significantly shorter latency to fall than WT-Veh mice (*p* = 0.0005, Tukey’s test). A strong trend for a decrease in this parameter was observed in FUS-tg-Ril mice compared to that in WT-Ril controls (*p* = 0.0552, Tukey’s test). Furthermore, the FUS-tg-POM16 group showed a significant increase in the latency to fall compared to vehicle-treated mutants (*p* = 0.0181, Tukey’s test; Figure 3G).

In addition, Fisher’s exact test showed a significant difference in the number of mice that fell during the first 10 s of the wire-hanging test between the groups (*p* = 0.0079). A trend was observed in the pairwise comparison of FUS-tg-Veh and FUS-tg-Ril mice (*p* = 0.0642, Fisher’s exact test; Figure 3H), whereas the comparison of FUS-tg-Veh and FUS-tg-POM16 mice showed a significant difference (*p* = 0.0244, Fisher’s exact test). Next, Fisher’s exact test revealed a significant difference in the percentage of immediate fall during the wire-hanging test between the groups (*p* < 0.0001). A significant difference was observed in the pairwise comparison of FUS-tg-Veh mice and FUS-tg-Ril mutants (*p* < 0.0001, Fisher’s exact test), as well as in the comparison of FUS-tg-Veh mice and FUS-tg-POM16 mice (*p* < 0.0001, Fisher’s exact test; Figure 3I).

In the open field test, two-way ANOVA revealed no significant main effects of genotype (F_1,80_ = 1.796, *p* = 0.1840) or treatment (F_2,80_ = 1.363, *p* = 0.2618), and no significant genotype × treatment interaction (F_2,80_ = 1.125, *p* = 0.3296) of the number of crossed sectors. FUS-tg-Veh mice crossed significantly fewer sectors than WT-Veh mice (*p* = 0.0488, Tukey’s test; Figure 3J). No significant differences between genotypes were observed in the riluzole-treated (*p* = 0.9489, Tukey’s test) or POM-16-treated groups (*p* = 0.6669, Tukey’s test). A strong trend for an increase in the number of crossed sectors was observed in FUS-tg-POM16 mice compared to FUS-tg-Veh mutants (*p* = 0.0531, Tukey’s test), while FUS-tg-Ril mutants did not differ significantly from FUS-tg-Veh mice (*p* = 0.2121, Tukey’s test).

Two-way ANOVA of the time spent in the center revealed significant main effects of genotype (F_1,80_ = 6.778, *p* = 0.0110) and a significant genotype × treatment interaction (F_2,80_ = 4.146, *p* = 0.0193), whereas the main effect of treatment was not significant (F_2,80_ = 0.5720, *p* = 0.5667). FUS-tg-Veh mice spent significantly less time in the center than WT-Veh mice (*p* = 0.0002, Tukey’s test). No significant differences between the genotypes were observed in the riluzole-treated (*p* = 0.6184, Tukey’s test) or POM16-treated groups (*p* = 0.8579, Tukey’s test). Furthermore, FUS-tg-POM16 mice showed a significant increase in the time spent in the center compared to vehicle-treated mutant mice (*p* = 0.0067, Tukey’s test; Figure 3K), whereas FUS-tg-Ril mutants did not differ significantly from FUS-tg-Veh mutants, although a trend was observed (*p* = 0.0907, Tukey’s test).

Finally, two-way ANOVA of the number of rearings revealed a significant main effect of genotype (F_1,80_ = 12.40, *p* = 0.0007), while no significant effects of treatment (F_2,80_ = 0.1469, *p* = 0.8636) or genotype × treatment interaction (F_2,80_ = 1.132, *p* = 0.3276) were observed. The FUS-tg-Veh group showed significantly fewer rearings than the WT-Veh controls (*p* = 0.0014, Tukey’s test; Figure 3L). Neither riluzole nor POM16 treatment significantly altered this parameter in the mutant mice (*p* > 0.05, Tukey’s test).

### 2.4. Histological Hallmarks of ALS Syndrome in FUS [1-359]-tg Mice and Effects of POM16

**Figure 4:**
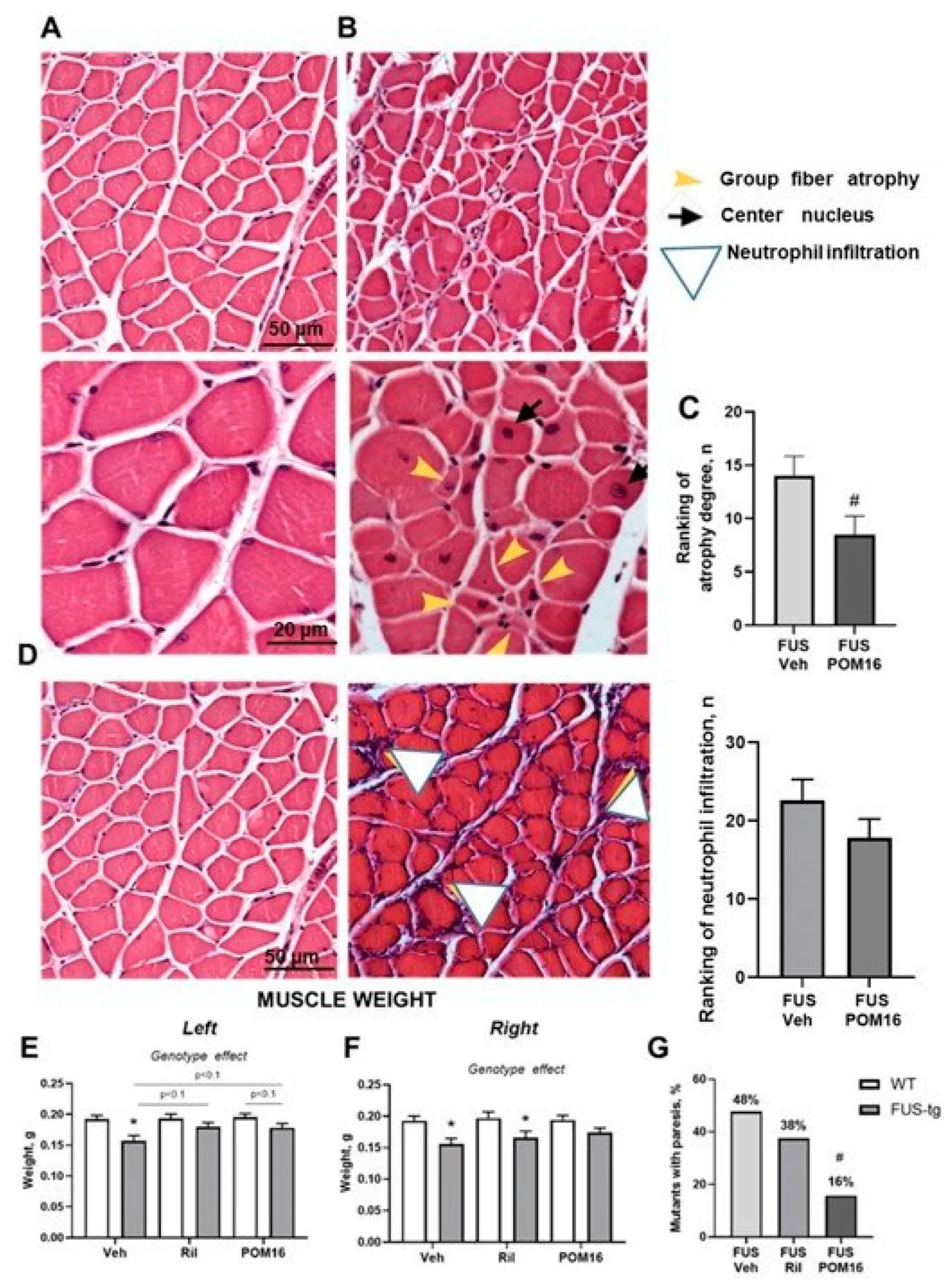
ALS-like muscle pathology in FUS [1-359]-transgenic mice treated with POM16 or riluzole. (**A**) WT mice displayed normal muscle morphology, while (**B**) FUS-tg animals showed characteristic for this model the presence of atrophic fibers and displaced cell nuclei. (**C**) Ranking score of the degree of muscle atrophy in mutant mice. (**D**) In addition, no signs of neutrophil infiltration were revealed in WT mice (**left image**), and pronounced neutrophil infiltration was found in mutants (**right image**). However, ranking analysis did not reveal significant differences between vehicle-treated and POM16-tretaed FUS-tg groups (*p* > 0.05, *t*-test, see ms text). (**E**) Average mass of the left and (**F**) right gastrocnemius muscles. (**G**) Percentage of mice with paresis evaluated on day 95 post-injury. Scale bar = 50 µm. * *p* < 0.05 vs. respective WT group; # *p* < 0.05 vs. FUS-tg-Veh group. Unpaired *t*-test, two-way ANOVA, post hoc Tukey’s test, and Fisher’s exact test. For muscle weight, 10 mice per group were used, and for atrophy scoring, 11 mice per group were used. Ril—riluzole. All data are mean ± SEM.

Morphological analysis of muscle tissue demonstrated the presence of atrophic fibers in FUS [1-359]-tg mice, which is characteristic of the ALS model used (Figure 4A,B). The unpaired *t*-test revealed that POM16 treatment significantly reduced the muscle gastrocnemius atrophy score in FUS [1-359]-tg mice compared to that in vehicle-treated mutant mice (*p* = 0.0425, t(20) = 2.166; Figure 4C). Another striking feature of the muscles of FUS [1-359]-tg mice was neutrophil infiltration (Figure 4D). Ranking analysis showed a trend towards lower neutrophil infiltration in POM16-treated mutants, while the group difference did not reach a significance level (*p* = 0.0963, t(20) = 1.326; Figure 4D).

Two-way ANOVA of the left gastrocnemius muscle weight showed a significant main effect of genotype (F_1,54_ = 14.19, *p* = 0.0004), whereas the main effect of treatment (F_2,54_ = 1.935, *p* = 0.1543) and the genotype × treatment interaction were not significant (F_2,54_ = 1.384, *p* = 0.2593). FUS-tg-Veh mice had significantly lower muscle weight than WT-Veh mice (*p* = 0.0009, Tukey’s test; Figure 4E). No significant differences between genotypes were observed in the riluzole-treated groups (*p* = 0.1974, Tukey’s test), while a trend toward a decrease in this measure was found in the FUS-tg-POM16 group compared to WT-POM16 mice (*p* = 0.0936, Tukey’s test). Both riluzole and POM16 treatment showed a tendency toward increased muscle weight in FUS-tg mice compared to the FUS-tg-Veh group (*p* = 0.0630 and *p* = 0.0976, respectively, Tukey’s test).

Two-way ANOVA of the right gastrocnemius muscle weight revealed a significant main effect of genotype (F_1,54_ = 17.52, *p* = 0.0001), whereas the main effect of treatment (F_2,54_ = 0.6584, *p* = 0.5218) and the genotype × treatment interaction did not reach statistical significance (F_2,54_ = 0.5045, *p* = 0.6066). Post hoc comparisons showed that FUS-tg-Veh animals had significantly lower muscle weights than WT-Veh animals (*p* = 0.0036, Tukey’s test). Similarly, this indicator was significantly decreased in the FUS-tg-Ril group relative to their WT counterparts (*p* = 0.0135, Tukey’s test; Figure 4F).

Importantly, Fisher’s exact test revealed a significant difference in the number of mice displaying paralysis at the age of 95 days (*p* < 0.0001, Figure 4G). While no significant difference was observed in the pairwise comparison of FUS-tg-Veh and FUS-tg-Ril mice (*p* = 0.1971, Fisher’s exact test), a comparison of FUS-tg-Veh and FUS-tg-POM16 groups showed a significant difference (*p* < 0.0001, Fisher’s exact test; Figure 4G).

**Figure 5:**
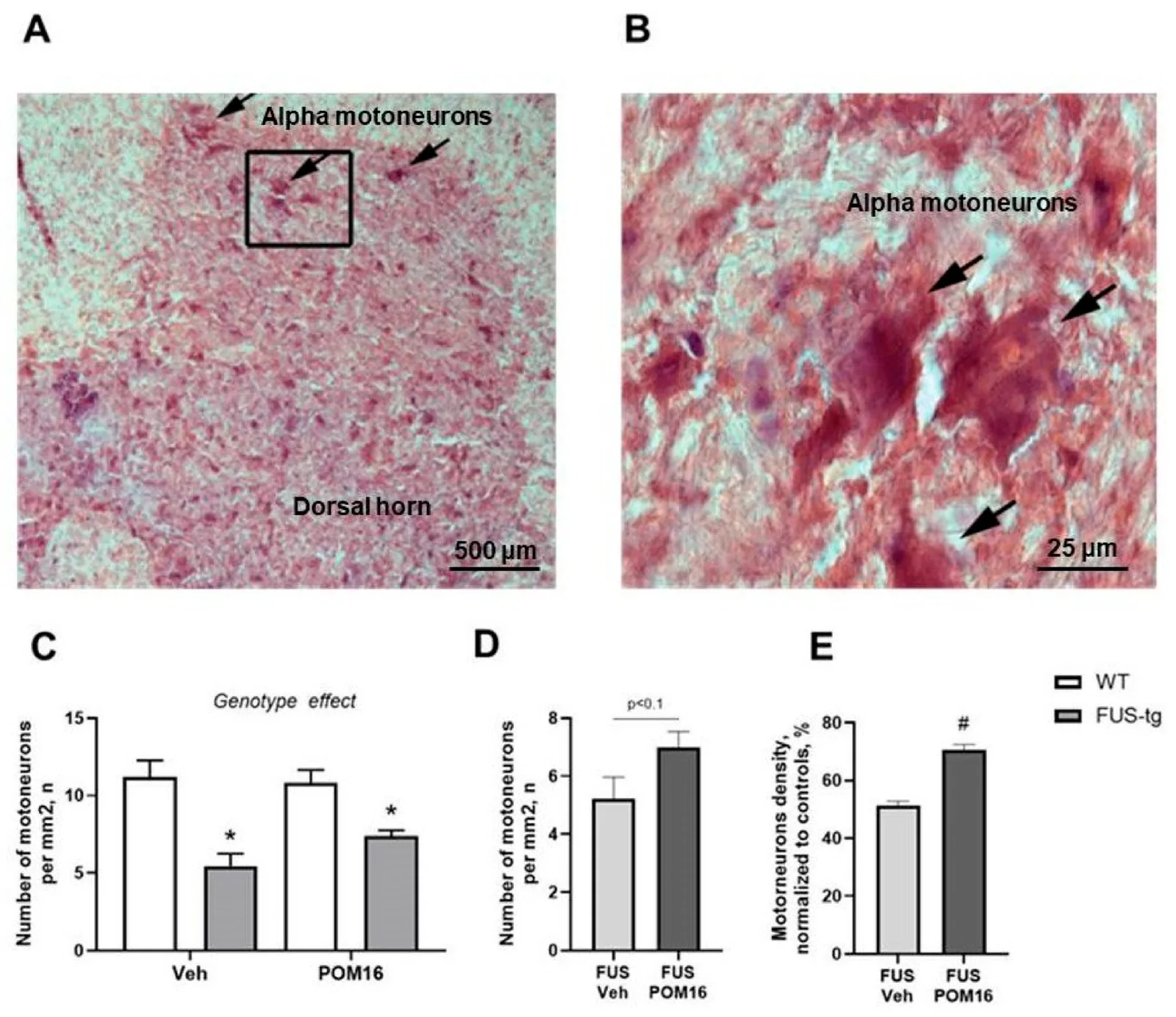
Spinal cord motoneuron density parameters in FUS [1-359]-transgenic mice treated with POM16 or riluzole. (**A**,**B**) Motoneuron morphology in FUS [1-359]-transgenic mice. (**C**,**D**) Number of motoneurons per mm^2^ of spinal cord area. (**E**) Motoneuron density was normalized to that of wild-type controls. * *p* < 0.05 vs. respective WT group, # *p* < 0.05 vs. FUS-tg-Veh group. Two-way analysis of variance (ANOVA) and post hoc Tukey’s test, unpaired *t*-test. For motoneuron counting, 5–9 mice per group were used in each experiment. Ril—riluzole. All data are mean ± SEM.

Nissl staining of spinal cord sections exhibited normal morphology in wild-type mice, with notable morphological alterations in FUS-tg mice (see Supplementary File, Figure S2), characterized by shrunken cell bodies and less defined nuclei (Figure 5A,B). Analysis of motoneuron density in the spinal cord using two-way ANOVA revealed a pronounced main effect of genotype (F_1,27_ = 28.15, *p* < 0.0001), whereas the main effect of treatment (F_1,27_ = 0.7561, *p* = 0.3922) and the genotype × treatment interaction (F_1,27_ = 1.840, *p* = 0.1862) failed to reach statistical significance.

A marked reduction in motoneuron number was detected in FUS-tg-Veh mice relative to WT-Veh animals (*p* < 0.0001, Fisher’s LSD test; Figure 5C). Similarly, FUS-tg-POM16 mutants displayed significantly fewer motoneurons than their WT counterparts (*p* = 0.0163, Fisher’s LSD test; Figure 5C).

Since POM16-treated animals revealed significant improvement in motor behavior and muscle histology in FUS-tg mice, the analysis of motoneuron density was performed in mutants treated with this compound or vehicle; an unpaired one-tailed *t*-test was used. FUS-tg-POM16 mice revealed a strong trend toward an increase in these measures compared to FUS-tg-Veh (*p* = 0.0873, t(14) = 1.838 and Figure 5D). A comparison of motoneuron density normalized to the WT-Veh group revealed a significantly higher motoneuron density in FUS-tg-POM16 mutant animals than in FUS-tg-Veh mice (*p* = 0.0274, t(13) = 2.109, *t*-test; Figure 5E). Histological comparison of WT-Veh, FUS-tg-POM16, and FUS-tg-Veh mice revealed a clear reduction in motoneuron density and deterioration of their morphology in the latter group (Supplementary File, Figure S2).

### 2.5. Molecular Changes in FUS [1-359]-tg Mice Treated with Riluzole or POM16

**Figure 6:**
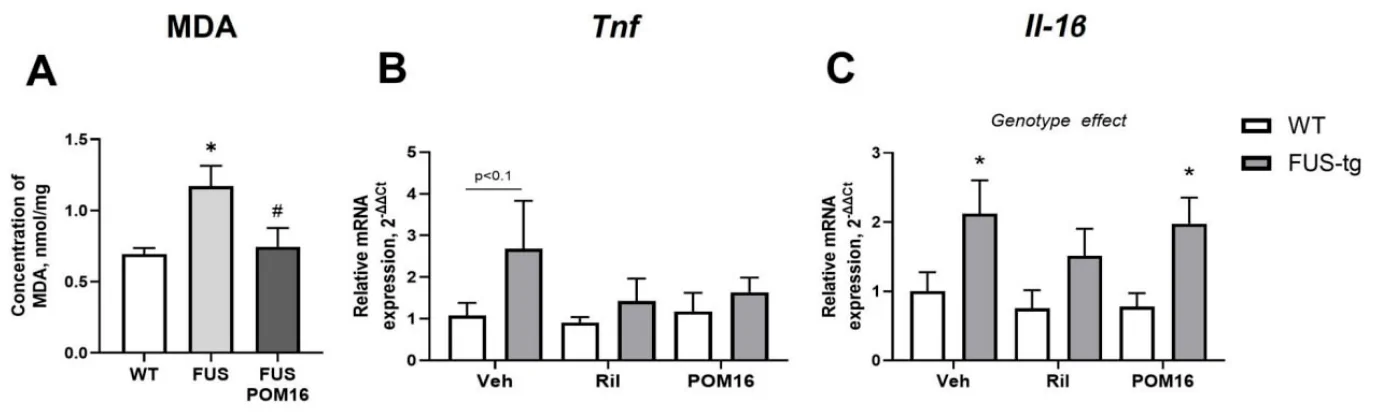
Effects of POM16 on the expression of molecular markers in FUS [1-359]-transgenic mice. (**A**) MDA levels, (**B**) *Tnf* expression, (**C**) *Il-1β* expression. * *p* < 0.05 vs. respective WT group, # *p* < 0.05 vs. FUS-tg-Veh group. One-way or two-way ANOVA, post hoc Tukey’s test. A total of 6–11 mice per group were used. Ril—riluzole. All data are mean ± SEM.

One-way ANOVA revealed significant group differences in MDA levels (F_2,30_ = 5.21, *p* = 0.0114). This indicator was significantly higher in FUS-tg-Veh mice than in WT-Veh controls (*p* = 0.0166, Tukey’s test). FUS-tg-POM16 mice showed significantly reduced MDA levels compared to FUS-tg-Veh mice (*p* = 0.0354; Figure 6A), bringing them to a level not significantly different from WT-Veh mice (*p* = 0.9443).

Two-way ANOVA of *Tnf* mRNA expression revealed no significant main effects of genotype (F_1,40_ = 2.225, *p* = 0.1436; Figure 6B) or treatment (F_2,40_ = 0.5669, *p* = 0.5717), and no significant genotype × treatment interaction (F_2,40_ = 0.4502, *p* = 0.6407). FUS-tg-Veh mice showed a trend toward elevated Tnf mRNA levels compared to WT-Veh controls (*p* = 0.0880, Tukey’s test), but this difference did not reach statistical significance.

Two-way ANOVA of *Il-1β* mRNA expression revealed a significant main effect of genotype (F_1,40_ = 10.61, *p* = 0.0023), while no significant effects of treatment (F_2,40_ = 0.6240, *p* = 0.5409) or genotype × treatment interaction (F_2,40_ = 0.1682, *p* = 0.8457) were found. FUS-tg-Veh and FUS-tg-POM16 mice showed significantly higher Il-1β mRNA levels than their respective WT controls (*p* = 0.0299 and *p* = 0.0363, respectively, Tukey’s test; Figure 6C).

## 3. Discussion

The present study describes the synthesis of a new compound designed as an isomer of the slow-releasing H_2_S donor L-thiovaline and reveals its protective activity in ameliorating experimental ALS syndrome in an established genetic model of this disease. Chronic dosing with POM16 at a relatively low dose of FUS [1-359]-tg mutants partially counteracted the neurodegeneration of motoneurons of the spinal cord, reduced muscular dystrophy, improved motor functions and general physical decline, and delayed the onset of paralysis. These effects were associated with the normalization of the oxidative stress marker MDA levels in the spinal cord. In line with commonly accepted guidelines [40], the effects of POM16 in FUS [1-359]-tg mice were compared with standard therapy with riluzole, whose partial therapeutic efficacy was demonstrated in current and previous studies with this model. Notably, the effects of POM16 were similar to or more pronounced than those elicited by standard treatment. Specifically, the administration of POM16, but not riluzole, significantly delayed paralysis in FUS [1-359]-tg mutants, precluded a loss of muscle weight and a reduction in liquid consumption; however, only riluzole-treated mutants showed normalized *Il-1β* gene expression in the spinal cord. While the mechanism of action of POM16 and its properties as a slow-releasing H_2_S donor require further investigation, it is remarkable that this small water-soluble molecule exerted significant effects of slowing down ALS-like neurodegeneration.

The FUS [1-359] mouse used in the present study is a commonly recognized animal paradigm of ALS [53] that reproduces a spinal, lower-limb-onset form of disease [3,4]. In these animals, the first overt motor sign is tremor and weakness of the hindlimbs, which is followed by hindlimb paresis and then by ascending paralysis, whereas forelimb and bulbar function remain relatively preserved until the terminal stage [51,81]. Therefore, motoneuron degeneration in this model is most pronounced in the lumbar spinal cord, and specifically among the motor neurons of the lateral motor column, which supply the distal hindlimb muscles, including the gastrocnemius [82]. This pattern corresponds to the limb-onset form of human ALS, which accounts for approximately two-thirds of all cases [83,84,85]. Accordingly, in the present study, the lumbar ventral horn and gastrocnemius muscle, to which motoneurons project from the lumbar section of the spinal cord, were chosen as the primary anatomical readouts of the disease process and its modification by treatment.

Following the generally accepted guidance in biomedical research using pre-clinical ALS models [40,86], we compared the effects of POM16 against the effects of riluzole applied in a previously validated dosing regime [58]. As expected, treatment with riluzole precluded some, but not all, disease-related physiological deficits [57,58]. The present data are in accordance with previously reported beneficial effects of riluzole in this genetic model of ALS, a dosing of which, based on 6-week riluzole administration via drinking water at the dose of 8 mg/kg/day starting at the pre-symptomatic age of the mutants of 8-9 weeks, prevented the significant decline in body weight and in food and liquid intake at weeks 5 and 6 of dosing in 11- to 12-week-old mutants, and counteracted motor deficits in the rotarod, pole, and wire tests [58]. Notably, the curative effects of this standard ALS therapy are only partial, which is consistent with clinical observations [8,87].

While the mechanisms of action and doses used cannot be compared to those of POM16, it should be noted that the effects of the latter on many evaluated readouts were similar to or greater than those of riluzole. Remarkably, the number of mutants displaying signs of paralysis (paresis) at the age of 95 days was significantly lower in the POM16-treated group than in the vehicle-treated group. Although riluzole-treated mutants revealed similar changes in this measure, the difference was not statistically significant. Specifically, the percentage of mice with paralysis on the 6th week of dosing was reduced from 48% in vehicle-treated mutants to 16% in POM16-treated FUS [1-359]-tg mice, while in the riluzole–treated group, it was 38%, which was not significant.

Notably, muscle weight was only partially improved by the latter treatment, unlike the dosing with POM16. In comparison with vehicle-treated FUS [1-359]-tg mice, POM16-treated mutants had significantly higher motor neuron density in the spinal cord, lower MDA levels, and reduced muscle atrophy ranking. Riluzole-treated mutants showed a significant loss of body weight normalized to control values only starting from week 5 of drug administration, and in POM16-treated mice, this difference was found in week 6 of treatment. Liquid intake measured at the end of the study was higher in FUS [1-359]-tg mice that received riluzole than in vehicle-treated mutants but was still significantly lower than that in the WT group, while POM16-treated FUS [1-359]-tg groups showed normal consumption. While both treatments ameliorated the motor functions of FUS [1-359]-tg mutants in the rotarod, wire, and pole tests, the latency to turn in this test was significantly ameliorated only in POM16-treated mutant mice.

In the rotarod model, the percentage of mice falling early in this test was significantly lower in the POM16-treated group but not in the riluzole-treated group of mutants. Similarly, in the wire test, the latency to fall was significantly increased in FUS [1-359]-tg mice that received POM16, but not in riluzole-treated mice, compared with pharmacologically naïve mutants. Since motor tests suggested greater effects of POM16 against the effects of rilozole, whose action on the muscles of the mutants was previously demonstrated, we limited our analysis to the former two groups of mice. Consistent with these findings, the POM16-treated FUS [1-359]-tg group exhibited a lower muscle atrophy score and higher density and number of motoneurons in the lumbar parts of the spinal cord. The preservation of motoneurons in this model by other treatments was previously associated with normalized muscle atrophy and motor function [57,58,59,60].

However, comparison of the latencies to fall in the rotarod test revealed no significant effects of treatment. In addition, although POM16-treated mutants exhibited a significantly higher motor neuron density than vehicle-treated FUS [1-359]-tg animals, this measure remained significantly lower than that observed in the control group, indicating that the therapeutic effects of POM16 were partial. Hence, beneficial action of POM16 does not fully reverse disease progression, reflecting the severe and multifactorial nature of ALS pathology [8,87].

The outcomes from other assays further suggest partial efficacy of the treatments used. Interestingly, we observed a trend towards lower neutrophil infiltration in POM16-treated mutants; while the group difference did not reach a significance level, this result can be considered as an additional hint to the potential anti-inflammatory effects of POM16. In line with this, while gene expression of TNF in vehicle-treated mutants revealed only a trend in the current study, the overexpression of this and other pro-inflammatory cytokines is a well-established feature of neurodegeneration in patients with ALS and FUS-tg mice [57,58,60,66]. Notably, mutants that received either treatment showed unaltered TNF expression, with levels close to the control values. However, the upregulation of another cytokine, IL-1β, in the FUS-tg vehicle-treated group was also observed in POM16-treated mice, but not in riluzole-treated FUS [1-359]-tg animals. The reasons for these discrepant changes remain to be investigated.

The present data can be seen as formally contradicting previously reported findings that ALS patients display elevated H_2_S levels, determined, however, using a uniquely developed HPLC modification method, and only in 30% of ALS patients with less common bulbar-onset clinical form of the disease [20]. The remaining cohort displaying upper limb upset showed H_2_S levels similar to those of healthy controls [20]. The same group showed increased H_2_S levels in neuronal cultures of SOD1G93A mutants and these were reversed by CBS inhibitor, which also exerted beneficial effects in these experiments [18,19,20]. However, H_2_S increase was only demonstrated in female mice and only at a specific time window described as the late phase of syndrome development, which is difficult to explain [20]. Notably, the current study used male mutants, which could explain the discrepant results from the previous report. Moreover, the reported beneficial effects of the H_2_S synthesis enzyme CBS inhibitor aminooxyacetate might be questionable, since CBS is not expressed in the spinal cord, where H_2_S production is governed by CSE [88]. In general, these results should be considered with caution because SOD1 utilizes metal ions at its active site, and metal ions such as Cu^2+^, Zn^2+^, and Fe^2+^ exhibit high affinity for H_2_S [89], which can bind to its catalytic center and increase enzyme activity under normal conditions [90]. In the context of ALS, for which significantly elevated levels of both free H_2_S and bound sulfur levels were found in the CSF of patients on one hand [19], and SOD1 mutations represent the most frequent causes, this interaction may potentiate pathological aggregation of enzymes and promote pathology.

The discrepancy between our findings and previously reported results may stem from several factors, apart from the sex of the animals used. First, as noted above, an earlier study was conducted using a single ALS paradigm based on an SOD1 mutation; SOD1 and H_2_S may interact with each other, suggesting that the results regarding H2S’s role obtained in relation to SOD1 mutation are gene-specific and potentially animal paradigm-specific. Second, as discussed above, CBS inhibition in these mice was applied at a stage corresponding to symptomatic disease, unlike our study. Next, the early molecular mechanisms underlying FUS- and SOD1-related pathology are substantially distinct, where FUS dysfunction leads to oxidative stress and inflammation at early stages of pathology, which are secondary pathological processes resulting from the accumulation of toxic aggregates of mutated gene products in the case of SOD1 gene mutation [1]. Moreover, the FUS [1-359]-tg model recapitulates features of the limb-onset form of ALS [49], in which H_2_S CSF levels were unchanged [19]. Thus, H_2_S regulation might differ between SOD1G93A and FUS [1-359]-tg mutants, which can lead to distinct results in the studies.

As such, it can be suggested that the effects of H_2_S supplementation may be dependent on whether or not SOD1 gene mutation is involved, and on the stage of disease progression [14,16,22,24]. During the early stages of ALS progression, corresponding to the presymptomatic period during which FUS [1-359]-tg mice received treatment in our study, increasing H_2_S availability—for example, through administration of an H_2_S donor such as POM16—may be beneficial owing to the antioxidant and anti-inflammatory properties of H_2_S. However, in the later stages of the disease, excessive H_2_S may exert detrimental effects, including the disruption of mitochondrial function [13,14,15,17]. Consistent with this interpretation, the present study demonstrated a reduction in MDA levels in the spinal cord of FUS [1-359]-tg mice following POM16 treatment, supporting the possibility that H_2_S supplementation exerts an antioxidant effect during the early stages of FUS-associated ALS pathology. Antioxidant activity and a suppression of MDA production in particular are well described effects of H_2_S donors [13]. Similarly, L-thiovaline was shown to enhance cGMP formation and to promote vasorelaxation in mouse aortic rings [91]. As prominent oxidative stress changes are characteristic of patients with ALS and animal models, including FUS [1-359]-tg mutants [6,7], an antioxidative stress action is likely to contribute to the beneficial effects of POM16. Our previous studies with FUS [1-359]-tg revealed the prominent beneficial effects of antioxidants, such as thiamine compounds, in this paradigm [59,60]. Finally, other potential pharmacological off-target effects of the POM16 compound may underlie the neuroprotective effects reported here and remain to be carefully investigated.

Generally, the available evidence regarding H_2_S dysregulation in ALS remains scarce and complex and is not yet fully understood. This complexity is further supported by reports of significantly reduced levels of H_2_S-bound sulfur species in the cerebrospinal fluid of patients with ALS [19], together with the absence of detectable changes in blood H_2_S levels [20], despite the high CSF levels of free H_2_S [20]. Conversely, H_2_S concentrations have been reported to decrease or increase in other neurological disorders [25,26,27,88].

Our work is the first step towards evaluating the potential therapeutic properties of the neuroprotective compound POM16 because of current limitations. H_2_S release remains to be studied in various tissues, while the slow-releasing properties of POM16 have been shown in bicarbonate buffer. Given that blood and intracellular medium contain bicarbonate at high (millimolar) concentrations, varying from 22 to 32 mM and 10 to 15 mM, respectively, thioamino acids have been suggested to exert H_2_S release in vivo [43]. Another limitation is currently lacking biochemical data on toxicity of POM16. However, as discussed previously, as thionorvaline is not implicated in protein synthesis [45], it is unlikely to exert toxic effects, allowing for the anticipation of good safety and tolerability of POM16.

Reported here, open field data indirectly suggest a lack of toxic effects of POM16 treatment, given a lack of group differences in total crossings that is further supported by a demonstration of absence of body weight loss in wild-type mice. On a separate note, as general locomotion was not affected by mutation in this assay, this rules out potential confounds in the evaluation of mouse behavior in motor tests. At the same time, vehicle-treated mutants showed increased anxiety-like changes, a previously described feature of these mice [57,66] as manifested by a diminished number of central crossings; a lack of these changes in both dosed FUS [1-359]-tg groups further indicates efficacy of the treatments used. Consistent with this, we observed a decrease in exploratory rearing activity in vehicle-treated FUS-tg mice, which was not observed in mutants treated with riluzole or POM16.

Thus, the new compound POM16 can be considered a compound with therapeutic potential for treating ALS pathology, likely mediated via antioxidative stress mechanisms. Given that any effective treatment of this devastating disease is currently lacking, it is hoped that POM16, a low-molecular-weight water-soluble molecule effective at low doses without signs of toxicity, may represent a target for more effective treatment of ALS disorder as a promising therapy for ALS.

## 4. Materials and Methods

### 4.1. Design, Synthesis and Characterization of 2S)-2-Aminopentanethioic S-Acid (POM16)

Carbonyl diimidazole (7.96 g; 49.12 mmol; CDI) was added to a solution of *N*-(tert- butoxycarbonyl)-(2S)-2-aminopentanoic acid (9.69 g; 44.65 mmol; compound **1**) in methylene chloride (60 mL) under stirring and cooling in an ice bath to obtain compound **2** (Figure 1). The reaction mixture was then stirred and maintained at room temperature for 1 h. After completion, an excess amount of H_2_S was passed into the reaction mixture while stirring and cooling in an ice bath for 3 h. Finally, 15 mL of 1M aqueous HCI was added to obtain compound **3**. The reaction mixture was concentrated under reduced pressure, diluted with ethyl acetate, cooled to 0 °C, and adjusted to pH 3. The organic layer was separated, washed with aqueous NaCl, dried with MgSO_4_, filtered, and concentrated under reduced pressure. Trifluoroacetic acid (20 mL, 262 mmol) was added, and the solution was stirred at room temperature for 2 h. After completion, the solution was concentrated under reduced pressure, and the white solid compound **4** ((2S)-2-aminopentanethioic S-acid) was isolated by filtration and dried in a vacuum desiccator for 72 h.

Yield: 47% of the starting compound **1**; white solids (2.80 g, 21.0 mmol). ^1^H NMR (400 MHz, DMSO-d6) δ ppm: 0.84 (t, *J* = 8.0 Hz, 3H); 1.31 (m, 2H); 1.63 (m, 1H); 1.81 (m, 1H); 3.45 (m, 1H); 7.76 (br 620 s, 3H). ^13^C NMR (100 MHz, DMSO-d6) δ ppm: 14.2; 18.4; 34.9; 62.4; 209.9. Molar rotation [M]_D_ + 77 (c = 0.4; water, 20 °C). Elemental analysis for C_5_H_11_NOS (molecular weight 133.21): calculated (%) C 45.08, H 8.32, N 10.52, S 24.07; found (%) C 44.86, H 8.19, N 10.46, S 23.90 (purity 98%).

Based on the adjustment of POM16 concentration to liquid intake, body mass, and compliance with a dosing procedure where solutions were freshly prepared twice per week, as well as the need to maintain a certain filling volume of the bottle, approximately 3 g of POM16 was used in the study, whereas the net calculated amount was 1.8 g.

### 4.2. Reagents Used in Synthesis

*N*-(tert-butoxycarbonyl)-(2S)-2-aminopentanoic acid, carbonyl diimidazole, and trifluoroacetic acid were obtained from Merck/Sigma-Aldrich (Darmstadt, Germany). The elemental compositions (carbon, nitrogen, hydrogen, and sulfur) were determined using a Eurovector EA-3000 analyzer (EuroVector S.p.A, Milan, Italy). ^1^H and ^13^C NMR spectra were recorded on a Bruker-400 spectrometer (Rheinstetten, Germany) using tetramethylsilane (TMS) as an internal reference and deuterated dimethyl sulfoxide (DMSO-d6) as a solvent. Chemical shifts (δ-scale) are expressed in parts per million (ppm) and coupling constants (J) in Hertz (Hz). The signal multiplicities were designated as follows: s (singlet), br s (broad singlet), t (triplet), and m (multiplet).

### 4.3. Generation of FUS [1-359]-tg Mice

Generation of FUS [1-359]-tg mice was performed as described elsewhere [49,51]. Briefly, a fragment of human FUS [1-359] cDNA including 9 bp of 5′-UTR was cloned into Thy-1 promoter plasmid 323-pTSC21k. A gel-purified fragment obtained by digestion of the resulting plasmid DNA with NotI was used for microinjection of mouse oocytes. Transgenic animals were identified by PCR analysis of DNA from ear biopsies by the presence of 255-bp product (primers 5′-TCTTTGTGCAAGGCCTGGGT-3′ and 5′-AGAAGCAAGACCTCTGCAGAG-3′). The original transgenic line on C57Bl6/CBA genetic background was backcrossed with CD1 wild-type mice for several (>7) generations.

### 4.4. Animals and Housing

A colony of FUS-tg mice and their wild-type littermates (WT) were bred in the FDA-certified SPF facilities of NRBSU, as described elsewhere [50,51]. Starting two weeks prior to the experiment, six-week-old male mice were single-housed under standard conditions (12 h light/dark cycle, lights on at 21:00, humidity 50–60%, temperature 22 ± 1 °C) with food and water ad libitum. All efforts were undertaken to minimize the potential discomfort of experimental animals. Experimental procedures were set up in accordance with a Directive 2010/63/EU and approved by the local veterinarian Committees for Bioethics of MSMU (22/10/17-MSMU-35; approved 22 October 2017). All efforts were undertaken to ensure compliance with the above-mentioned regulations concerning human endpoints in animal research that were established before the start of the study. The animals were closely monitored throughout the study. Therefore, the mice were examined at least twice daily starting at of 85 days. Humane endpoints included severe motor dysfunction, that is, inability to right within 30 s and inability to reach food or water, or showing severe weight loss (>20%). Animals meeting these criteria were excluded from the experiment, euthanized using an overdose of isoflurane, followed by cervical dislocation, in accordance with institutional ethical approval.

### 4.5. Study Flow with FUS [1-359]-tg Mice

The experimental design was based on the reported pattern of pathology development in the FUS [1-359]-transgenic mouse model of ALS [57,58,59] and in compliance with the requirements for any proof of concept and preclinical studies [40]. The course of the ALS-like pathology in this genetic model is that following the pre-symptomatic phase [57,66], typically lasting until the age of 3 months, FUS [1-359]-tg mice mutants start displaying rapidly progressing paresis leading to the human endpoint within approximately 2 weeks [50,51,58,60]. Other potential confounding variables were systematically controlled.

**Figure 7:**
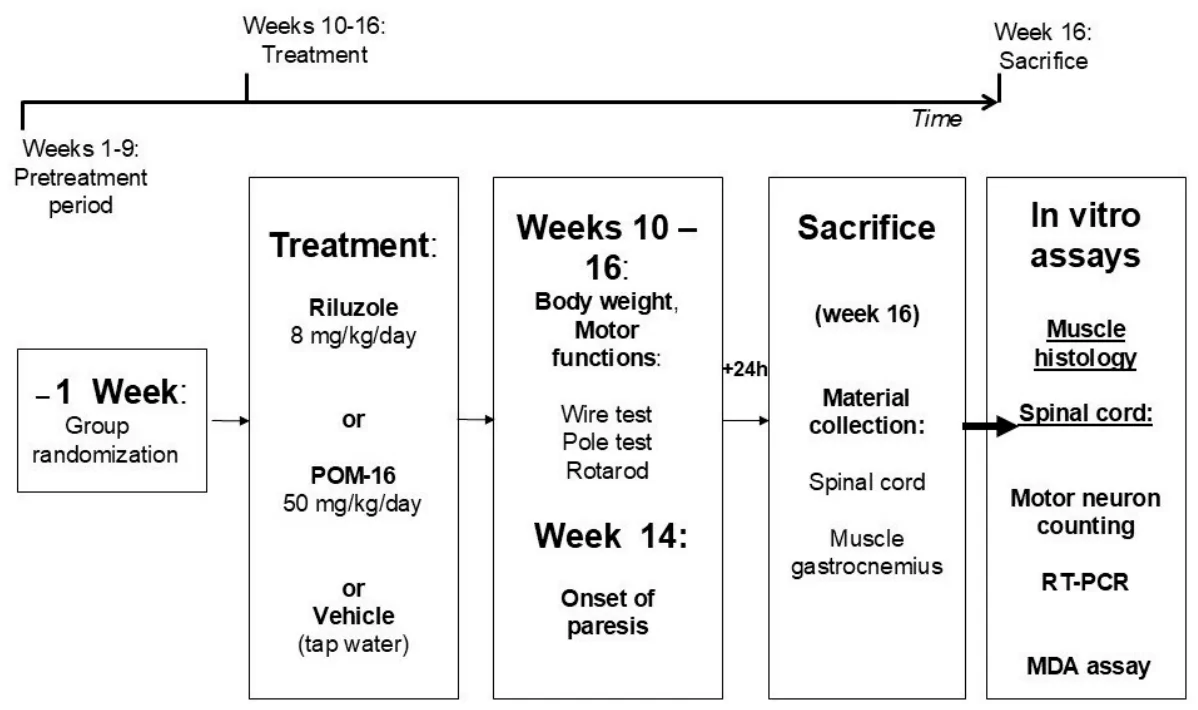
Experimental design of in vivo experiments with FUS-treated Tg mice. FUS [1-359]-Tg mice and their WT littermates were divided into vehicle-, riluzole and POM16- treated groups. Mice received treatment from the age of nine weeks; motor function was tested in week 5, followed by the open field test; at the age of 95 days, mutants were assessed for a presence of paralysis (paresis). At week 6, all groups were studied for liquid and dietary intakes. After 24 h, the mice were euthanized, and the lumbar region of the spinal cord was collected and fixed for histological analysis and molecular assay. The gastrocnemius muscle was dissected, weighed, and fixed for histological analysis.

At the age of nine weeks, groups of male FUS [1-359]-tg mice and wild-type (WT) littermates were randomized for the upcoming treatment according to their body weight and date of birth (Figure 7). The administration of POM16 (at a dose of 50 mg/kg/day) was provided to 19 mutants and 10 WT mice, treatment with riluzole (at a dose of 8 mg/kg/day) was realized for 16 mutants and 10 WT animals, and 23 FUS-tg and 10 WT littermates received vehicle (tap water). Total number of animals used in the study was 88. The sample size was determined based on previous studies [57,58,59,60,66], with the aim of balancing statistical power with ethical considerations. Dosing was performed by replacing drinking water with treatment solutions, as described elsewhere [57,58,59,60,66]. The substances were administered for a total period of 6 weeks, and body weight was monitored weekly. In week 5, the motor behavior of all animals was scored using the wire test, pole test, and rotarod test, as described elsewhere [57,58,59,60,66]. An open field test was also performed. In the same week, at the age of 95 days, the onset of paralysis was recorded in the groups of FUS-tg mutants, and the percentage of mice displaying paralysis was calculated as previously established parameters of disease progression and effects of treatments [59,60]. At week 6, 24 h diet intake and 12 h liquid intake were evaluated. At the end of this week, all mice were euthanized, and their spinal cords and gastrocnemius muscles were harvested for further histological and molecular analyses. This analysis included the MDA assay and RT-PCR of pro-inflammatory cytokines. In addition, the left and right gastrocnemius muscles were weighed (Figure 7). The experimenter remained blind to the group assignments until the data analysis phase.

### 4.6. Physiological Readouts and Motor Scores

Weekly measured body weights of mutant mice were normalized to the weights of the WT group according to the treatment received (Figure 7). Body weights during weeks 1–6 were evaluated. On the 6th week, 24 h food intake and 12 h water intake were evaluated by weighing the bottles, as described elsewhere [92].

### 4.7. Rotarod Test

Mice were placed on a constantly rotating rod of rotarod (Columbus Instruments, Columbus, OH, USA; speed 10 rpm) for 600 s. The latency to fall and the number and percentage of mice with falling events (latency < 200 s) were registered in three runs, as described elsewhere [57,58,59,60].

### 4.8. Pole Test

Animals were placed on top of a vertical bar (diameter 1.1 cm, height 60 cm) and allowed to climb down to a horizontal surface. The latency to descend the bar and the number and percentage of mice with sliding events were scored as described previously [57,58,59,93,94]. In addition, we recorded the latency of turning on mice on the top of the wooden bar as the time between the placement of a mouse there and the start of a descent.

### 4.9. Wire Test

Mice were allowed to grip a horizontal wire (diameter 0.3 cm, height above the surface 60 cm) for 180 s. The latency of falling and the number and percent of mice with falling events (latency < 10 s) were recorded in each group as described elsewhere [57,58,59,93,94].

### 4.10. Open Field

All mice were investigated in the open field test using an apparatus (45 × 45 × 45 cm, Technosmart, Rome, Italy) under illumination intensity of 25 lx. The animals were positioned in the corner of the arena and monitored for 5 min. The number of crossed sectors (5 × 5 cm each), duration of time spent in the center of the arena (15 × 15 cm) and the number of rearings were scored offline using automated analysis with a software (ViewPoint, v.5, Civrieux, France) as previously described [93,94,95].

### 4.11. Preparation of Drug Solutions and Administration of Drugs

Potential effects of POM16 were compared to effects of riluzole that can be dissolved and delivered with drinking water. Drugs were continuously administered with drinking water because the half-life of POM16 in mice was found to be approximately one hour [46]. In addition, previous studies have revealed the partial therapeutic efficacy of this method for riluzole, which resembles its therapeutic activity in the clinic [57,58,59,60,67,87]. Riluzole tablets (Sandoz, Almere, The Netherlands) were crushed and dissolved in tap water; the concentration was adjusted to the dosage of 8 mg/kg/day and daily water intake in mice as described elsewhere [57,58].

### 4.12. Recording of the Onset of Paralysis

Starting from age 85 days, mutant mice were examined twice a day for a manifestation of signs of paresis; age at which paresis was first detected, was recorded. In the preceding time period mutants were observed every 2–3 days, so as WT groups during entire duration of the study.

### 4.13. Killing of Mice and Tissue Collection

Mice were terminally anesthetized using CO_2_ and isoflurane, following the previously established protocols [94,95,96]. Both gastrocnemius muscles were dissected and weighed and placed into 4% paraformaldehyde. The lumbar parts of the spinal cord were collected as described elsewhere [58,59] and placed in 4% paraformaldehyde. The torocical parts of spinal cords were dissected, rapidly frozen in dry ice, and stored at −80 °C until needed for MDA and PCR assays.

### 4.14. Scoring for Muscle Atrophy and Neutrophil Infiltration

Muscle gastrocnemius from FUS-tg mice that were treated with vehicle or POM16 was fixed, sectioned and stained for hematoxylin and eosin as described elsewhere [58]. Scoring for atrophy was performed by three independent pathologists, blinded to sample identity using a light microscope (Axiovision 4.3, Zeiss, Berlin, Germany); ranking histograms were generated, ranking samples from 1 to 40, from “moderate” (1) to “severe” (40) atrophy as described elsewhere [58]. Likewise, ranking analysis was performed for scoring of muscle infiltration with neutrophils by two experimenters blind for group identification. Histological samples were ranked from 1 to 40 as (1) “not infiltrated” to (40) “highly infiltrated”.

### 4.15. Motoneuron Counting

Fixed lumbar parts of the spinal cord were cross-sectioned at a thickness of 50 μm as described elsewhere [58]. The cuts, encompassing L3–L5 with 250 μm intervals, were cut and stained with thionine NISSL (Sanova, Hamburg, Germany). Counts were performed by three observers blinded to sample identity using a Zeiss Axoplan2 system (Zeiss, Berlin, Germany).

### 4.16. Quantitative Real-Time PCR (qRT-PCR)

Total mRNA was extracted from each sample of spinal cord of mutant and WT mice using the RNeasy Lipid Tissue Mini Kit (Qiagen, Hilden, Germany). For first-strand cDNA synthesis, 1 μg of total RNA was reverse-transcribed into cDNA using the QuantiTect Reverse Transcription Kit (Qiagen, Hilden, Germany), following the previously established protocols [93,94,95]. qRT-PCR was conducted with the SYBR Green master mix (Bio-Rad Laboratories, Philadelphia, PA, USA) on a ProFlex PCR system (Thermo Fisher Scientific, MA, USA). Each reaction (10 μL) contained 5 μL SYBR Green master mix, 3 μL RNase-free water, 1 μL of specific forward and reverse primers (20 pmol/μL), and 1 μL cDNA. *β-actin* was chosen as the reference gene due to its relatively stable expression in the brain, as previously reported [94,95,96]. The qRT-PCR protocol included an initial denaturation at 95 °C for 4 min, followed by 40 cycles of denaturation at 95 °C for 20 s and annealing at 54 °C for 90 s. Primer sequences are listed in Table 1. All samples were run in triplicate. Data were normalized to *β-actin* mRNA expression and calculated as relative fold changes, following established methods [94,95,96].

### 4.17. Malondialdehyde (MDA) Assay

The levels of MDA in the spinal cord tissue were assessed using an Enzyme-linked Immunosorbent Assay Kit for MDA (Cloud-Clone Corp., Houston, TX, USA) according to the manufacturer’s instructions, as described elsewhere [97,98]. Brain tissue was weighed before analysis and homogenized using a handheld rotor-stator homogenizer TissueRuptor II (QIAGEN Sciences Inc., Germantown, MD, USA). The absorbance of MDA compounds generated from lipid peroxidation was measured at 450 nm using a microplate reader Feyond-A400 (Allsheng, Hangzhou, China).

### 4.18. Statistical Analysis

Data were analyzed using GraphPad Prism software version 6.00 (GraphPad Software, San Diego, CA, USA). Depending on the number of groups, multiple group comparisons were analyzed using one-way, two-way, and repeated-measures ANOVA, followed by Tukey’s test. The Mann–Whitney test was used for two-group comparisons. Ranking analysis was performed using a *t*-test. All datasets were tested for normal distribution to choose the correct statistical method. Qualitative data were analyzed using Fisher’s exact test. No data points were excluded from the analysis. The level of significance was set at *p* < 0.05. The results are presented as bars with standard errors of the mean (SEM).

## 5. Conclusions

Given the high medicinal need to find an effective remedy for ALS patients and the body of evidence showing the role of H_2_S in this disorder, a new low-weight water-soluble isomer of the slow-releasing H_2_S donor thiovaline POM16 was designed, synthesized, and studied for its potential interference with ALS-like syndrome in mice. Our study provides the first evidence for the therapeutic efficacy of POM16 as a treatment option capable of partially counteracting the progression of ALS-like pathology and accompanying oxidative stress. In comparison with the standard pharmacological reference rilzole, this compound induced a greater rescue effect on the onset of LS syndrome and motor and physiological parameters. Further research is needed to elucidate the mechanisms of pharmacological action and other effects of POM16 on the hallmarks of ALS, including its potential as a slow releaser of H_2_S in vivo. Despite these and other limitations, POM16 may be considered a potential therapeutic remedy for patients with ALS.

## Figures and Tables

**Table 1:** Sequence of primers used.

Gene	Forward Sequence	Reversed Sequence
*β-actin*	CACTGAGCATCTCCCTCACA	CACTGAGCATCTCCCTCACA
*Tnf*	GGGAGCAGAGGTTCAGTGAT	TTGTCTTAATAACGCTGATTTGGT
*Il-6*	TAGTCCTTCCTACCCCAATTTCC	TTGGTCCTTAGCCACTCCTTC

## Data Availability

Data are available on appropriate request via T.S or I.P.

## Supplementary Materials

The following supporting information can be downloaded at https://www.mdpi.com/article/10.3390/molecules31173021/s1, Supplementary File (Figure S1 Kinetics of H_2_S release induced by (2S)-2-aminopentanethioic S-acid (POM16), Figure S2 Microphotographs of cross-sections of the ventral horns of the lumbar segment of spinal cord from the wild-type and FUS [1-359]-tg mice treated with vehicle or POM16.).

## Abbreviations

The following abbreviations are used in this manuscript:

ALS	Amyotrophic lateral sclerosis
H_2_S	Hydrogen sulfide
FUS	Fused in sarcoma
SOD1	Superoxide dismutase 1
SOD1G93A	Superoxide dismutase 1 Gly93Ala mutant
CBS	Cystathionine β-synthase
CSE	Cystathionine γ-lyase
3-MST	3-Mercaptopyruvate sulfurtransferase
MDA	Malondialdehyde
POM16	Novel slow-releasing hydrogen sulfide donor isomer
TNF	Tumor necrosis factor
IL-1β	Interleukin-1β
GSK-3β	Glycogen synthase kinase-3β
Iba-1	Ionized calcium-binding adapter molecule 1
nNOS	Neuronal nitric oxide synthase
cGMP	Cyclic guanosine monophosphate
NaHS	Sodium hydrosulfide
Na_2_S	Sodium sulfide
H_2_O_2_	Hydrogen peroxide
MPTP	1-Methyl-4-phenyl-1,2,3,6-tetrahydropyridine
6-OHDA	6-Hydroxydopamine
WT	Wild-type
Tg	Transgenic
qRT-PCR	Quantitative real-time polymerase chain reaction
mRNA	Messenger RNA
cDNA	Complementary DNA
ANOVA	Analysis of variance
SEM	Standard error of the mean
FDA	Food and Drug Administration
CSF	Cerebrospinal fluid
BSS	Bound sulfur species
NLS	Nuclear localization signal
Boc	tert-Butoxycarbonyl
CDI	Carbonyl diimidazole
DMSO-d6	Deuterated dimethyl sulfoxide
NMR	Nuclear magnetic resonance
HPLC	High-performance liquid chromatography
SPF	Specific pathogen-free
LSD	Least significant difference
