# Supplementary material for: Beneficial Effects of Putative Hydrogen Sulfide (H2S) Donor POM16 in a Genetic Model of Amyotrophic Lateral Sclerosis, FUS [1-359]-Transgenic Mice
Source: Molecules. 2026 Aug 28;31(17):3021. doi: 10.3390/molecules31173021 (PMC13567389; doi:10.3390/molecules31173021)
Supplement: Supplementary file 1 [file molecules-31-03021-s001.zip › molecules-4491301-supplementary.pdf]

Supplementary File

1.Study of stability of (2S)-2-aminopentanethioic S-acid (POM16)

The stability of POM16 was studied at cold and room temperatures. During the 16-week period, solid compound POM16 did not undergo significant changes, accounting for less than 2% at 2–8°C, as determined by elemental analysis before the start and after the termination of the experiment. A solution of POM16 in water did not undergo significant changes during storage at 25°C for one week, as determined by comparing the 1H NMR spectra. As the storage solid compound was kept at +4 °C and solutions for in vivo study were prepared every 2-3 days to be used freshly, we assume that available stability data suggest a possibility of degrading POM16 during dosing of mice to be very unlikely.

2.Study of kinetics of H_2S release by (2S)-2-aminopentanethioic S-acid (POM16)

We studied the kinetics of H_2S release from (2S)-2-aminopentanethioic S-acid (POM16) using the established H_2S donor, Na_2S, as a reference compound. The H_2S concentration was measured using a commercially available cell-permeable, highly specific, sensitive, and fast-responsive S fluoroprobe H_2S probe P3 (Sigma-Aldrich, Darmstadt, Germany). H_2S detection was based on the rapid reaction between P3 and H_2S (Singha et al., 2015). Fluorescence was excited at 387 nm. The emission was recorded at 520 nm wavelength. (2S)-2-aminopentanethioic S-acid (100 μM) or 100 μM Na_2S was added to bicarbonate buffer (10 mM, pH 7.4) at 25°C in the presence of P3, and fluorescence was monitored for one hour every 5 min. The results for the POM16 samples were measured in five replicates and normalized to the values obtained for the Na_2S-treated samples. We found that under employed conditions, (2S)-2-aminopentanethiic S-acid (POM16) induced the release of H_2S in bicarbonate buffer (Fig.S1). As expected, this effect was weaker than that of Na_2S, a fast-releasing H_2S donor.

Figure S1

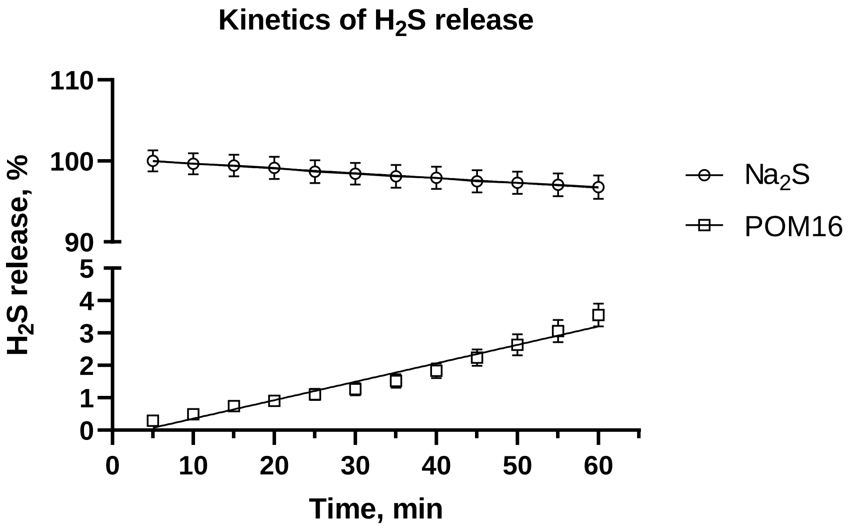

Supplementary Figure S1. Kinetics of H_2S release induced by (2S)-2-aminopentanethioic S-acid (POM16). In bicarbonate buffer (10 mM) at pH 7.4 and 25°C, a slow release of H_2S was induced by POM16. The results are presented as the percentage of H_2S concentration from the values of the reference Na_2S-treated sample. Data are shown as Mean ± SEM (n = 5 replicates).

3. A comparison of motoneuron density in the spinal cord of wild type and FUS[1-359]-tg mice.

For Nissl staining (Lysikova et al., 2019), the sections were deparaffinized three times for 5 min each in xylene and rehydrated at room temperature in a series of alcohols according to the following scheme: 96% alcohol for 5 min, 90% alcohol for 2 min, and 75% alcohol for 2 min; they were then incubated for 2 min in deionized water with the addition of three drops of glacial acetic acid. For staining, the sections were placed for 10 min in a solution of cresyl violet (Santa Cruz, CA, USA), rinsed quickly with 75% alcohol, and differentiated in 96% ethanol containing 0.25% glacial acetic acid. Dehydration of the sections was performed in a series of alcohols with increasing concentrations, cleared with xylene, and embedded in the synthetic medium BioMount (BioOptica, Italy) using the automatic embedding station Leica CV5030 (Leica Biosystems, Berlin, Germany). Representative microphotographs of the lumbar segment of the spinal cord from the wild-type and FUS[1-359]-tg mice (segments 3-5) treated with vehicle or POM16 show a reduction in motoneuron density in the latter mice (Fig.S2).

Figure S2

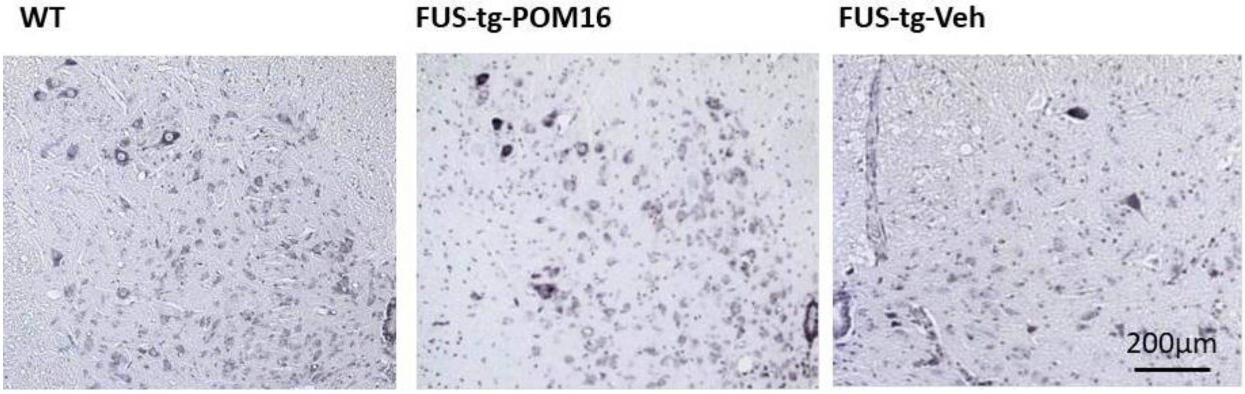

Supplementary Figure S2. Microphotographs of cross-sections of the ventral horns of the lumbar segment of spinal cord from the wild type and FUS[1-359]-tg mice treated with vehicle of POM16. Note greater number of motoneurons and their preserved histology in FUS-tg-POM16 mouse in comparison with FUS-tg-Veh mouse. There was visible reduction of motoneuron number and detoriation of cellular structure in FUS-tg mice in comparison with WT. WT – wild type mice, FUS-tg - FUS[1-359]-tg mice, Veh – vehicle, POM16 (see ms text) **M**agnification ×100, bar 200 μm.
